# RIS-Assisted Multi-Antenna AmBC Signal Detection Using Deep Reinforcement Learning

**DOI:** 10.3390/s22166137

**Published:** 2022-08-16

**Authors:** Feng Jing, Hailin Zhang, Mei Gao, Bin Xue, Kunrui Cao

**Affiliations:** 1 School of Telecommunication Engineering, Xidian University, Xi’an 710126, China; 2 School of Information and Communication, National University of Defense Technology, Xi’an 430035, China; 3 Shaanxi Key Laboratory of Intelligence Coordination Networks, Xi’an 710048, China; 4 Faculty of Electronic and Information Engineering, Xi’an Jiaotong University, Xi’an 710049, China

**Keywords:** reconfigurable intelligent surface, ambient backscatter communication, signal detection, multi-antenna, deep reinforcement learning

## Abstract

Signal detection is one of the most critical and challenging issues in ambient backscatter communication (AmBC) systems. In this paper, a multi-antenna AmBC signal detection method is proposed based on reconfigurable intelligent surface (RIS) and deep reinforcement learning. Firstly, an efficient multi-antenna AmBC system is developed based on RIS, which can achieve information transmission and energy collection simultaneously. Secondly, a smart twin delayed deep deterministic (TD3) AmBC signal detection method is presented, based on deep reinforcement learning. Extensive quantitative and qualitative experiments are performed, which show that the proposed method is more compelling than the outstanding comparison methods.

## 1. Introduction

In recent years, with the development of modern digital society, new services and applications such as smart cities, smart houses, digital Internet of Everything, and wired/wireless distributed sensing systems have been constantly emerging, putting forward significantly higher requirements for communication capability [1].

Surprisingly, ambient backscatter communication (AmBC) has been one of the most promising paradigms for the modern communication technologies [2]. Particularly, the reciprocal communication among energy-free equipment is achieved with backscattering radio frequency (RF) signals [3,4]. Different from the traditional backscatter systems [5], the requirements for a part of dedicated infrastructures are weakened with the appearance and growth of AmBC. Furthermore, the exploration of the scarce and expensive wireless spectrum is improved by the available AmBC signals. Particularly, AmBC signal detection is a fundamental and difficult problem to be solved.

So far, there has been some research focused on AmBC signal detection that can be roughly divided into three categories: the physical methods [6], the statistical methods [7], and the learning methods [8]. However, there are several common thorny problems on most of the approaches above: (1) the obtained parameters are incomplete; (2) the direct link interference is serious; (3) the hide intrinsic representations and characteristics are difficult to extract. These issues above make it difficult for the most existing AmBC signal detection methods to be applied well in practice. Therefore, efficient and practical AmBC signal detection methods are urgently needed in both the theoretical and industrial fields.

It is worth noting that deep learning, especially deep reinforcement learning (DRL), has been widely used in wireless communications [9,10,11]. Learning rules are ingeniously developed, and intrinsic features are learned autonomously from the environment by DRL, which is difficult to obtain by traditional methods in wireless communication systems. It has been proven that DRL may be an effective way to perform the design, operation, and optimization of the reconfigurable intelligent surface (RIS)-based wireless communication systems.

Therefore, in this paper, the RIS-assisted multi-antenna AmBC signal detection is investigated, and an efficient signal detection method is proposed based on DRL and RIS. Specifically, the contributions of this work can be summarized as follows:

(1) An RIS-assisted multi-antenna AmBC signal model is developed, which can achieve information transmission and energy collection cooperatively.
(2) A twin delayed deep deterministic (TD3) AmBC signal detection method is developed based on deep reinforcement learning and reconfigurable intelligent surface.
(3) Extensive quantitative and qualitative experiments are performed, showing that the presented method is more compelling than the state-of-the-art comparison approaches.

This paper is organized as follows. The related studies are given in Section 2. The AmBC system model and the problem formulation are shown in Section 3. The proposed solutions are described in Section 4. The experimental results, comparisons, and analysis are presented in Section 5. The conclusions are offered in Section 6.

## 2. Related Works

Hu et al. [12] proposed a signal detection method for a backscatter communication system in a supervised learning manner, where the label signal of AmBC is detected by transforming the detection problem into a classification problem. Particularly, support vector machine and random forest are used to decode label symbols. Simultaneously, efficient features are extracted to minimize the bit error rate of the AmBC system. Experimental results show that with different extended gains, the proposed detector has a lower bit error rate and higher data rate than the traditional minimum mean squared error (MMSE) detectors at low signal-to-noise ratio.

Wang et al. [13] proposed an AmBC signal detection system in the binary phase shift keying (BPSK) modulation based on machine learning. Particularly, BPSK-based backscattering signal encoded by Hadamard code can be decoded with this method. Firstly, the direct path signal is eliminated, the residual signal is associated with the rough environment signal estimation to extract the tag signal’s learnable features. Then, the *k*-nearest neighbor classification algorithm is used to recover the tag signal. The recovered signal is decoded by Hadamard to obtain the original information bits. Finally, the effectiveness of the method is verified by experimental simulation.

In reference [14], the AmBC signal detection problem is transformed into a clustering problem, and two kinds of known labels are transmitted from the tags as prior knowledge to assist cluster initialization and signal detection. By directly using the received signals, two clustering-based detection methods are developed, i.e., one is the clustering of labeled signals (CLS), and the other one is the clustering of labeled and unlabeled signals (CLUS). Both two methods are developed based on the proposed modulation constrained Gaussian mixture model (GMM). Compared with the optimal detection method with perfect correlation channel state information, the proposed method has only a small gap.

An AmBC signal detection method is constructed in reference [15] in an unsupervised learning manner. The characteristics of the received signals are directly utilized, which are clustered with an unsupervised learning means. In addition, the tag bits are transmitted for cluster bit mapping to assist signal detection without the estimation of channel coefficients and noise power. For the spread spectrum gain *N* > 1 and *N* = 1, two detection methods are proposed, in which the features follow different mixed distributions. The detection threshold is derived with the learned parameters to optimize the detection performance. Finally, a large number of simulation results are given to verify the performance of the proposed scheme.

In reference [16], the tag signal detection problem for AmBC systems were studied by adopting the deep transfer learning (DTL) technology. Firstly, a universal DTL-based tag signal detection framework is designed, which uses a deep neural network (DNN) to implicitly extract the features of communication channels and directly recover the tag symbols. Based on the established pre-trained DNN and a few pilots, a DTL-based likelihood ratio test (DTL-LRT) was obtained through transfer learning. Moreover, exploiting the advantages of the convolutional neural networks’ powerful capability in exploring features of data in a matrix form, a covariance matrix-aware neural network (CMNet) for the sample covariance matrix is designed, and a CMNet-based detection algorithm is proposed. Finally, the simulation shows that the proposed CMNet method can achieve a close-to-optimal performance without explicitly obtaining the channel state information.

There are some differences between our proposed method and [12,13,14,15,16]; the main differences are as follows. 

First, supervised learning is used in references [12,13,14,16], manual intervention is required during learning, and the operation is complex and time-consuming. While in the proposed method, the unsupervised learning manner is applied, which does not need manual intervention, and the parameter learning is performed according to the data and model structure; thus, it is easy to operate. Moreover, the proposed method does not require a large number of manual annotation data in advance, so complex environments can be actively explored and better solutions can be obtained. 

Second, for [12], it is difficult to deal with missing data and the multi-classification problem, which can be handled well through deep reinforcement learning in our proposed method. Moreover, the relationships between data are easy to be ignored in [12]. While for the proposed method, the explicit data, implicit data, and the relationship between data can be explored. For [13], an appropriate proximity measure *K* and data preprocessing are required; otherwise, wrong predictions can be achieved by *k*-nearest neighbor. While this problem does not exist in our proposed method, which is simple, convenient, and flexible. For [14], before signal detection using the CLS and CLUS clustering methods, the signal needs to be preprocessed with the modulation-constrained GGM, which is inflexible and has difficulty dealing with the situations different from agglomerative hierarchical clustering. While in the proposed method, pre-modulation is not needed, and both the same and different agglomerative hierarchical distributions can be processed. For [15], unsupervised learning is used, which also does not need to label the data, but RIS is not used to improve the channel. The difference of signal characteristics when sending 0 and 1 tags is relatively small, which influences the improvement of detection effect. For [16], an AmBC signal detection method is proposed based on a deep transfer learning framework and convolutional neural network, so the requirement of channel state information computation is eliminated. The problem of insufficient annotation data is solved. However, a large amount of pre-training, fine-tuning, and maul intervention are required, which is relatively complex and not flexible enough. While in our proposed method, pre-training, fine-tuning, and maul intervention are not needed. 

Moreover, compared with references [12,13,14,15,16], RIS and multi-antenna are introduced into AMBC signal detection in our proposed method; furthermore, the signal detection problem is converted into the optimization problem of the RIS phase shift matrix and receiving antenna combination coefficient, and then deep reinforcement learning without data labels is presented to effectively solve the optimization problem, which improves the signal detection performance of AmBC system. 

## 3. System Model and Problem Formulation

### 3.1. Scenario Definition

**Figure 1:**
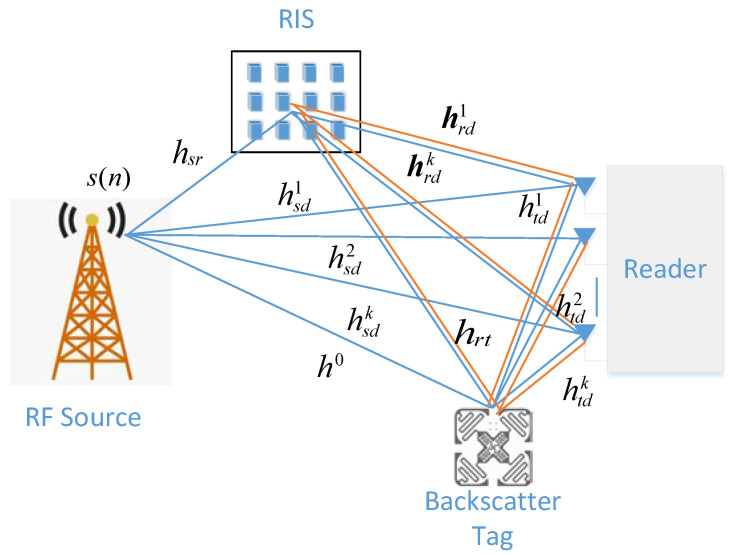
RIS-assisted AmBC system.

As shown in Figure 1, a downlink AmBC system assisted by RIS is described, which consists of an ambient radio-frequency (RF) source with single antenna, a reader equipped with K(K≥1) antennas, a tag equipped with single antenna, and a RIS with N(N≥1) reflectors [17]. The RF signal to the backscatter tag is offered by the ambient RF source, while its own information is transmitted. The backscatter tag’s information is modulated by on-off modulation to the ambient RF signal. A large number of reflective elements and passive beamforming are used to enhance the tag-to-reader wireless transmission link, while counteracting the direct link from ambient RF sources to the reader at the RIS. Multiple antennas are utilized at the reader to linearly combine the received signals to enhance the signal’s reception ability. 

In this section, the flat-fading channel is assumed. The channel from the ambient RF source to the tag, from the RF source to the k-th antenna of reader, from the RF source to the n-th reflector of the RIS, from the backscatter tag to the k-th antenna of the reader, from the tag to the n-th reflector of RIS, and from the n-th reflector of RIS to the k-th antenna of the reader, is denoted by $h_{0}\in {\mathbb{C}}^{1\times 1}$, $h_{sd}={\left[\begin{array}{cccc} h_{sd}^{1} & h_{sd}^{2} & \cdots & h_{sd}^{k} \end{array}\right]}^{T}\in {\mathbb{C}}^{K\times 1}$, $h_{sr}^{}={\left[\begin{array}{cccc} h_{sr}^{1} & h_{sr}^{2} & \cdots & h_{sr}^{n} \end{array}\right]}^{T}\in {\mathbb{C}}^{N\times 1}$, $h_{td}={\left[\begin{array}{cccc} h_{td}^{1} & h_{td}^{2} & \cdots & h_{td}^{k} \end{array}\right]}^{T}\in {\mathbb{C}}^{K\times 1}$, $h_{rt}^{}={\left[\begin{array}{cccc} h_{rt}^{1} & h_{rt}^{2} & \cdots & h_{rt}^{k} \end{array}\right]}^{T}\in {\mathbb{C}}^{N\times 1}$, $h_{rd}^{k}={\left[\begin{array}{cccc} h_{rd}^{1} & h_{rd}^{2} & \cdots & h_{rd}^{n} \end{array}\right]}^{T}\in {\mathbb{C}}^{N\times 1}$ and $H_{rd}^{}=\left[\begin{array}{cccc} h_{rd}^{1} & h_{rd}^{2} & \cdots & h_{rd}^{k} \end{array}\right]\in {\mathbb{C}}^{N\times K}$, respectively. Assume that all the channels follow the independent and identically distributed circularly symmetric complex Gaussian (CSCG) random variables with a mean value of zero and a variance of $E\left[{\left|h_{k}\right|}^{2}\right]=\sigma_{h}^{2}$. Assume also that the channel state information (CSI) at the reader is not known, which is consistent with the actual situation. In practice, due to the complexity of the device, the channel often cannot be observed by the reader.

### 3.2. Problem Description

According to the above scenario, the equivalent complex baseband signal sent by the ambient RF source is denoted as s(n). The signals reach the tag by the reflected RF source-RIS-tag path and the direct RF source-tag path, which is denoted by the symbol x(n), and can be obtained by the following formula:

(1) $$x(n)=\left(h_{0}+h_{sr}^{H}\Phi h_{rt}\right)s(n)$$

where $\Phi =\mathrm{diag}\left(e^{j\theta_{1}},e^{j\theta_{2}},\cdots ,e^{j\theta_{N}}\right)\in {\mathbb{C}}^{N\times N}$ denotes the phase shift matrix of the RIS, and $\theta_{n}\in \left[0,2\pi \right)$ for n=1,2,⋯,N is the phase shift induced by the *n*-th reflect element. The tag can be switched between reflection and non-reflection by adjusting the impedance, so that two kinds of information “0” and “1” can be carried, which is also called on-off modulation. The tag is a passive device that cannot achieve the complete reflection due to the material limitations, and hence there will be a certain attenuation during reflection. Therefore, after being modulated by backscatter, the transmitted signal of the tag can be expressed as

(2) t(n)=ηd(l)x(n), d(l)=0 or 1

where η represents a plural number indicating the attenuation of the tag, d(l) denotes a 1-bit effective information that the tag needs to send to the reader. The tag information’s symbol rate is often lower than that of the ambient RF source, due to the limitations in tag equipment and energy consumption. Therefore, assume that *L* RF sources symbols are spanned in 1 backscattered tag symbol. Thus, *L* satisfies the following relationship as:

(3) $$L=\frac{T_{tag}}{T_{source}}$$

where $T_{source}$ and $T_{tag}$ are the duration of the RF source and tag symbol, respectively.

### 3.3. Multi-Antenna Receiver Design

In general, the AmBC receiver owes larger physical size and more power and thus can support multiple antennas receiving together and more complex receiving algorithms than the AmBC tag [18]. In addition, due to the insurmountable BER floor problem in a single antenna, while the spatial diversity can be used with multiple antennas to effectively improve the system’s transmission performance, the multiple antennas at the backscatter receiver are exploited for joint reception.

Signals from the RF source and the tag are received at the reader. There are two components in the signal from the RF source, i.e., one is the direct RF source-receiver link, and the other one is the reflected RF source-RIS-receiver link. Furthermore, there are also two components in the signal from the tag; i.e., one is the direct tag-receiver link, and the other one is the reflected tag-RIS-receiver link. Thus, for the *k*-th receiver antenna, the received signal $r_{k}(n)$ can be expressed as:

(4) $$r^{k}(n)=H_{sd}^{k}s(n)+H_{td}^{k}t(n)+u^{k}(n),$$

where $H_{sd}^{k}=h^{k}+h_{sr}^{H}\Phi h_{rd}^{k}$, $H_{td}^{k}=h_{td}^{k}+h_{tr}^{H}\Phi h_{rd}^{k}$, and $u^{k}(n)∼CN(0,\sigma_{}^{2})$ denotes the equivalent noise at the receiver. The received signal at all *K* receiver antennas is expressed as $r\left(n\right)={\left[r_{}^{1}(n)\;r_{}^{2}(n)\;\cdots \;r_{}^{K}(n)\right]}^{T}$. Separating the two cases of d=0 and d=1, the received signal r(n) can be written as:

(5) $$r\left(n\right)=\{\begin{array}{ll} H_{sd}^{}s(n)+u(n) & ,d=0\\ H_{sd}^{}s(n)+H_{td}^{}t(n)+u(n) & ,d=1 \end{array}.$$

Taking Equations (1) and (2) into Equation (7), the following equation can be achieved:

(6) $$r\left(n\right)=\{\begin{array}{ll} H_{1}s(n)+u(n) & ,d=0\\ \left(H_{1}+H_{2}\right)s(n)+u(n) & ,d=1 \end{array}$$

where

(7) $$H_{1}=\left(h+h_{sr}^{H}\Phi h_{rd}^{}\right)$$

(8) $$H_{2}=\eta \left(h_{td}^{}+h_{tr}^{H}\Phi h_{rd}^{k}\right)\left(h_{0}+h_{sr}^{H}\Phi h_{rt}\right)$$

Finally, the received signals on the multiple antennas are combined by a linear combiner at the receiver. The combine coefficient is represented by the vector $f\in {\mathbb{C}}^{K\times 1}$, generally, ${\left\|f\right\|}^{2}=1$ without loss of generality. Therefore, the received signal after the combiner can be expressed as $y\left(n\right)=f^{H}r\left(n\right)$. 

### 3.4. Energy Detection

After the combiner, energy detection is then used to recover the signal transmitted from the backscatter tag. According to the proposed RIS AmBC system model, both s(n) and u(n) follow the circularly symmetric complex Gaussian (CSCG) distribution, and the received signal r(n) and the combined signal y(n) also follow CSCG distribution, which can be express as:

(9) $$y\left(n\right)∼\{\begin{array}{c} CN\left(0,W_{0}\right),d=0\\ CN\left(0,W_{1}\right),d=1 \end{array}$$

where

(10) $$W_{0}=f^{H}H_{1}H_{1}^{H}f+\sigma_{}^{2}I$$

(11) $$W_{1}=f^{H}\left(H_{1}+H_{2}\right){\left(H_{1}+H_{2}\right)}_{}^{H}f+\sigma_{}^{2}I$$

According to [17], the energy detector is the optimal detector to estimate the information of d:

(12) $$Z=\frac{\rho }{L}\sum_{n=1}^{L}{\left|y(n)\right|}^{2}\overset{d=1}{\underset{d=0}{≷}}\frac{\rho W_{0}W_{1}}{W_{1}-W_{0}}\ln\frac{W_{1}}{W_{0}}$$

where $\rho =\mathrm{sgn}(W_{1}-W_{0})$. As shown in Equation (12), when d=0, the mean of Z is $\mu_{0}=W_{0}$, and the variance of Z is $\sigma_{0}^{2}=W_{0}^{2}/N$. When d=1, the mean of Z is $\mu_{1}=W_{1}$, and the variance of Z is $\sigma_{1}^{2}=W_{1}^{2}/N$. In the case of very large *N*, applying the central limit law, the BER for backscatter tag signal detection can be obtained when ρ>0 as follows:

(13) $$P_{e}=\{\begin{array}{c} \frac{1}{2}Q\left(\frac{T_{h}-\mu_{0}}{\sigma_{0}}\right)+\frac{1}{2}Q\left(\frac{\mu_{1}-T_{h}}{\sigma_{1}}\right),\;\mathrm{when}\;\rho >0\\ \frac{1}{2}Q\left(\frac{T_{h}-\mu_{1}}{\sigma_{1}}\right)+\frac{1}{2}Q\left(\frac{\mu_{0}-T_{h}}{\sigma_{0}}\right),\;\mathrm{when}\;\rho <0 \end{array}$$

where $T_{h}=\frac{\mu_{0}\mu_{1}}{\mu_{1}-\mu_{0}}\ln\frac{\mu_{1}}{\mu_{0}}$.

### 3.5. Problem Formulation

Assume that γ denotes the maximum energy difference of the received symbols, $\gamma =\max\left(\frac{\mu_{1}}{\mu_{0}},\frac{\mu_{0}}{\mu_{1}}\right)$, and γ is brought into Equation (13). Thus the two cases of Equation (13) can be combined as:

(14) $$P_{e}=\frac{1}{2}Q\left(\sqrt{L}\left(\frac{\gamma }{\gamma -1}\ln\gamma -1\right)\right)+\frac{1}{2}Q\left(\sqrt{L}\left(1-\frac{\ln\gamma }{\gamma -1}\right)\right)$$

As shown in Equation (14), the BER of the AmBC system decreases monotonically with γ increasing. This is consistent with the real situation. As γ increases, the gap between $\mu_{0}$ and $\mu_{1}$ becomes greater. Thus, the parts of the received signals’ conditional probability distributions under d=0 and d=1 are greater. So, the area of overlap between the two probability distribution curves is smaller, which means that the BER is smaller. 

In this section, our goal is to maximize the maximum energy ratio γ by jointly optimizing the active beamforming vector at the reader and the passive phase shift of the RIS, subjected to the RIS phase constraint, as well as the energy limitation of the beamforming vector. Mathematically, this problem can be formulated as follows:

(15a) $$\;\;P1:\underset{f\;\;\theta }{\max}\;\gamma$$

(15b) $$s.t.\left|\Phi_{i}\right|=1,\;\;\;\;\forall i=1,2,\cdots ,N$$

(15c) $${\left\|f\right\|}^{2}=1$$

Since the objective function and constraints are non-convex, **P1** is a non-convex optimization problem. Generally, the alternating optimization (AO) method is used to solve the fixed weighting coefficient problem, but there is relatively high complexity. Furthermore, for the problem **P1**, it is difficult for AO to handle the weighting coefficient’s dynamic change. In this article, a DRL framework is proposed to solve the problem **P1** effectively.

## 4. Proposed Solution

In this section, for the problem above, the proposed DRL-based optimization problem solution is detailed. First, the progress of DRL is summarized, and then the proposed TD3 algorithm is given. 

### 4.1. Overview of Deep Reinforcement Learning

Deep reinforcement learning is the combination between reinforcement learning and deep neural network [19]. A conventional DRL system consists of agents, environment, and the mutual transfer of information between them constituting a Markov process. The current state $s_{t}$ of the environment is obtained through the agent, then the action $a_{t}$ is taken based on the policy Π and it is transmitted to the environment; $a_{t}$ is evaluated by the environment to send reward $r_{t}$, and the new state is updated to $s_{t+1}$. With the goal of maximizing long-term returns, deep neural networks and the accumulated empirical data are combined in the agent to continuously optimize the policies. So far, three value-based algorithms, including DQN, DDPG, and TD3, have emerged successively.

(1) DQN: Deep Q network (DQN) aims to solve the optimization problem of the discrete action space [20]. A Q function is constructed with a deep neural network to evaluate the action-state pair, which is expressed as Q(s,a;θ) and is updated by the maximum value of the Q function as Equation (16). However, Equation (16) cannot be used to solve the continuous action space problem well.
  (16) $$Q\left(S,A\right)\leftarrow Q\left(S,A\right)+\alpha \left[R+\gamma \underset{a}{\max}Q\left(S^{\prime },a\right)-Q\left(S,A\right)\right]$$
(2) DDPG: To solve the untractable problem that DQN cannot deal with the continuous action space, deep deterministic policy gradient (DDPG) is proposed [21]. There are two important components in DDPG: an Actor network and a Critic network. In the Actor network, a deep neural network is used to generate a deterministic policy $\mu \left(s;\theta_{\mu }\right)$ and then select the optimal action from the continuous action space, which can obtain the maximum value of Q. In the Critic network, $Q\left(s,a;\theta_{q}\right)$ is used to evaluate the action’s effect as the same as DQN. To solve the problem that the updated target continues to change when the network is updated, which makes the updating difficult, fixed network technology is used to generate the copies of the Actor network and the Critic network as the target network, and to generate the target value. The target network is softly updated by the following formula:
  (17) $$\theta_{i^{\prime }}=\tau \theta_{i}+\left(1-\tau \right)\theta_{i^{\prime }},\;i=\mu \;\mathrm{or}\;q$$
  Since a certain policy is generated by the Actor network, fully exploring the environment, the noise $N_{a}∼N\left(a_{t},1\right)$ is added to μ to overlap a random sample of normal distribution with $a_{t}$ as the mean. Here, the new policy $\tilde{\mu }$ can be expressed as
  (18) $$\tilde{\mu }\left(s_{t}\right)=\mu \left(s_{t};\theta_{\mu }\right)+N$$
(3) TD3: The Twin Delayed Deep Deterministic (TD3) policy gradient algorithm is an optimized version of DDPG [22]. DDPG is mainly improved by TD3 through three aspects: double Q network, delayed actor update, and the target policy smoothing regularization.
  **Target policy smoothing regularization:** When the target policy network outputs actions, noise following standard normal distribution is added, so that the estimation of Q is more accurate and the network is more robust. The output action is denoted as $\tilde{a}$, which is expressed by
  (19) $$\tilde{a}=\Pi_{\varphi^{\prime }}(s^{\prime })+\varsigma$$
  where ς∼clip(N(0,σ),−c,c) represents the normal noise with its upper and lower limits ±c.
  **Double Q network:** Two sets of networks are designed in TD3 to estimate Q, and the smaller one is regarded as the update target. The update target Q network is denoted as $y_{t}$ and expressed as follows:
  (20) $$y_{t}\leftarrow r_{t}+\gamma \min_{i=1,2}Q_{\theta_{i}}(s_{t+1}^{\prime },\tilde{a})$$
  where $s_{t+1}^{\prime }$ denotes the next state in minibatch, ${\tilde{a}}_{t}$ is the action after smoothing regularization, γ is the discount factor, $r_{t}$ is the reward what we designate at the current state.
  **Delayed actor update:** Delay here means the time delay of the actor network updating, that is, the actor is updated after the critic is updated several times. This is because the actor network’s goal is to achieve the maximum *Q* value, and the critic network generating the *Q* value is constantly updated. Therefore, it is difficult for the actor networks to find the optimal *Q* value, or even get stuck in the sub-optimal value. By introducing a delay, the actor network is updated when the *Q* value of the critic network is relatively determined. The actor network is updated by the following equation:
  (21) $$\theta_{i}\leftarrow -\min_{i=1,2}\sum {\left(y_{t}-Q_{\theta_{i}}(s_{t}^{\prime },a^{\prime })\right)}^{2}$$
  where $\left(s_{n},a_{n}\right)$ is the state-action pair from replay buffer and n=1,2,⋯D, D is the size of the replay buffer.

The target critic network and the actor network are soft-updated by the following equations, respectively:

(22) $$\theta_{i}^{\prime }\leftarrow \tau \theta_{i}^{}+(1-\tau )\theta_{i}^{\prime }\;$$

(23) $$\varphi_{i}^{\prime }\leftarrow \tau \varphi_{i}^{}+(1-\tau )\varphi_{i}^{\prime }$$

### 4.2. TD3-Based Framework

**Figure 2:**
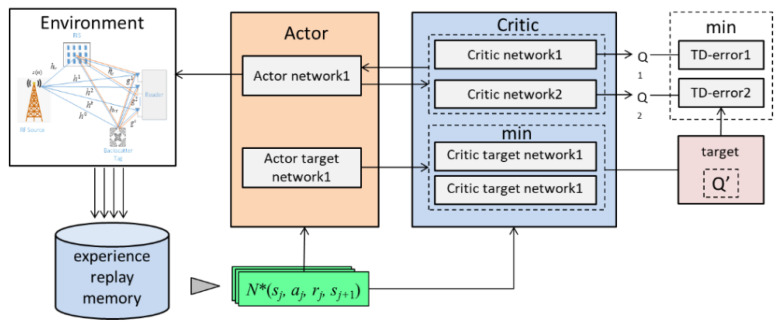
The TD3-based framework.

Analyzing the above algorithm, DQN is not suitable to solve the problem **P1**, because DQN can only handle the discrete action spaces. It is worthy that both the active beamforming and the passive RIS phase shift are continuously variable. While DDPG is easily influenced by the overestimation of the *Q* value, the optimal performance cannot be achieved, and DDPG is very sensitive to the external conditions. In this paper, an optimization algorithm based on TD3 is proposed to solve the non-convex optimization in problem **P1**, and the block diagram of TD3 is shown in Figure 2. We used six fully connected deep neural networks (DNNs) to build the algorithm, containing one Actor network, one Actor target network, two Critic networks, and two Critic target networks. Each DNN network contains one input layer, two hidden layers and one output layer.

(1) Construction of TD3: In the paper, the AmBC system and the RIS controller are regarded as the environment and the agent, respectively. In order to perform the TD3 algorithm, the state, action, and reward are defined as follows:
  **State space:** The state space represents the current state of the system, which consists of the optimization parameters of the previous time. Since the optimization parameter f denotes complex number value, and the neural network cannot handle the complex number, f is decomposed into two parts: the real part $f^{\prime }$ and the imaginary part $f^{*}$, and they are propagated to the neural network at the same time.
  (24) $$S_{t}=\left[f^{\prime }{}_{1}^{\left(t-1\right)},\ldots ,f^{\prime }{}_{K}^{\left(t-1\right)},f_{1}^{*\left(t-1\right)},\ldots ,f_{K}^{*\left(t-1\right)}\right]$$
  **Action space: There are two key components in the** action space: the combining vector f and the phase shift ϕ. Similarly, each complex variable is decomposed into the real and the imaginary part.
  (25) $$A_{t}=\left[\phi^{\prime }{}_{1}^{t},\ldots ,\phi^{\prime }{}_{N}^{t},\phi_{1}^{*t},\ldots ,\phi_{N}^{*t},f^{\prime }{}_{1}^{t},\ldots ,f^{\prime }{}_{K}^{t},f_{1}^{*t},\ldots ,f_{K}^{*t}\right]$$
  **Reward:** In this article, the optimization goal is to achieve the maximum energy ratio γ. The reward at step t is a linear transformation of maximum energy ratio γ, i.e.,
  (26) $$r^{t}=100(\gamma -1)$$
(2) Algorithm Description: The detail of the TD3 based framework is shown as follows (Algorithm 1):

**Algorithm 1** The TD3-based detector for AmBC1: **Input:** The batch size, the soft update coefficient, the replay capacity, the policy 2: network learning rate, the *Q* network learning rate, the policy target update interval,3: the explore steps, the action noise for exploration and evaluation4: **Output:** The optimal phase shift matrix
$\phi_{opt}$, the combining vector $f_{opt}$, the optimal
5: energy ratio *γ*, and the BER for backscatter tag detection6: **Initialization:** Randomly initialize the six networks7: **for** episode
j=1,…,M
**do**
8:            Obtain the current CSI9:            Randomly chose combining vector to obtain $s_{0}$10:            **for** step t=1,…,T
**do**
11:            **if**
*t* > exp *lore steps*, choose action based on the policy $s_{t}=\mu (s_{t};\theta_{\mu })+N$
12: **else** generate action randomly13:            Obtain the reward $r_{t}$ and observe new state $s_{t+1}$ by the environment, store
14: the tuple $\left\{s_{t},a_{t},r_{t},s_{t+1}\right\}$ into the replay memory
15:            **if** the length of replay buffer > batchsize, sample a minibatch from the 16: : experience replay memory17:            Learn and update the Critics network by minimizing the loss18:            **if** reach the policy target update interval, update the actor policy network19:            Update the target network parameters of Actor and Critics network20:            **end for**21: **end for**22:            Obtain the optimal phase shift matrix $\phi_{opt}$ and the combining vector $f_{opt}$ by final action $A_{t}$. Obtain the AmBC BER according to Equation (14) by the final $r^{t}$

It is worth noting that in practical systems, due to the limitations of the physical properties of the device, continuous phase shifts are approximated by multi-bit quantization of discrete phase shifts at the present [23,24,25]. For this purpose, the method proposed in this paper is still valid and only requires quantization of the optimal phase shift output to the nearest discrete phase shift. Of course, we can also replace the TD3 agent that solves continuous non-convex optimization with a DQN agent for discrete states and actions within the framework proposed in this paper. 

## 5. Numerical Results and Analysis

In this section, extensive quantitative and qualitative experiments, analysis, and discussion are performed to demonstrate the effectiveness of the proposed TD3 signal detection method for RIS-based multi-antenna AmBC. 

### 5.1. Experimental Implementations 

The related critical simulation parameters are set as follows: (1) all the RIS channels undergo Rician fading with Rician factor 3; (2) all other channels experience Rayleigh fading; (3) the path loss exponent is 2.5 for all the channels; (4) the ambient signal frequency is 2.4 GHz with transmit power 20 dBm; (5) the initial phase shift of the RIS and the initial received combined vector are set randomly and then are optimized gradually with TD3; (6) the critic and actor networks are fully connected deep neural networks (DNNs), which have one input layer, one output layer and two hidden layers; (7) the size of the actor and critic nets are (2*K*, 2*N* + 2*K*, 4*N* + 4*K*, 2*N* + 2*K*) and (2*N* + 4*K*, 4*N* + 8*K*, 2*N* + 4*K*, 1), respectively. The DNN parameters are shown in Table 1. 

The simulated performance is achieved by performing the average of 5000 independent channels. All the training is executed by Windows 10 and CPU with 11th Gen Intel(R) Core(TM) i7-1165G7 @ 2.80 GHz.

### 5.2. The Effect of the Number of Reflectors

**Figure 3:**
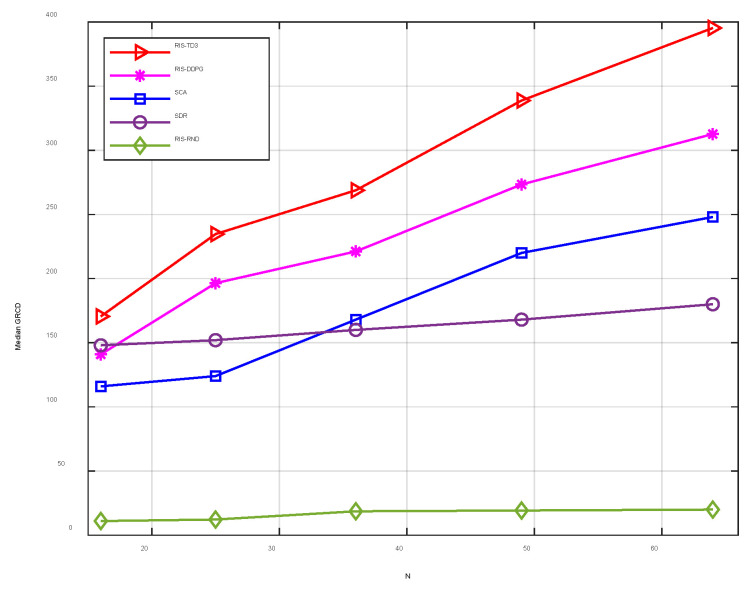
Median GRCD versus the number of antenna *N* at the reader with *K* = 4, Hidden_dim = 128.

In this section, the proposed TD3-based RIS-assisted multi-antenna AmBC signal detection method (denoted as RIS-TD3) is simulated to evaluate the effect of the receiving antennas number and the RIS reflective elements on the maximum energy ratio. The comparison benchmark algorithms include: (1) the RIS-assisted DDPG method, denoted as RIS-DDPG; (2) the RIS-assisted conventional method to optimize the phase shifts at the RIS, where the successive convex appropriation (SCA) method is used [26] denoted as SCA; (3) the semi-define relaxation (SDR) method that is compared [27] denoted as SDR; (4) the random RIS phase shift and receiver combiner coefficients, denoted as RIS-RND. The ambient RF source’s transmit power and the ambient noise variance are set as 20 and −95 dBm, respectively. The simulation results are illustrated in Figure 3.

Figure 3 shows the median GRCD versus the number of antennas at the reader with different algorithms. It is observed from Figure 3 that the proposed RIS-TD3 algorithm significantly outperforms the DDPG and the conventional methods. Particularly, with the increased antenna number, the three algorithms’ system performance can be enhanced, while the benefit of the proposed TD3 algorithm is the most important. This is because both the phase shift of RIS and the beamforming at the receiver are optimized by TD3, hence achieving the overall optimization, which obtains better median GRCD.

### 5.3. The Effect of Communication Distance on System Performance

**Figure 4:**
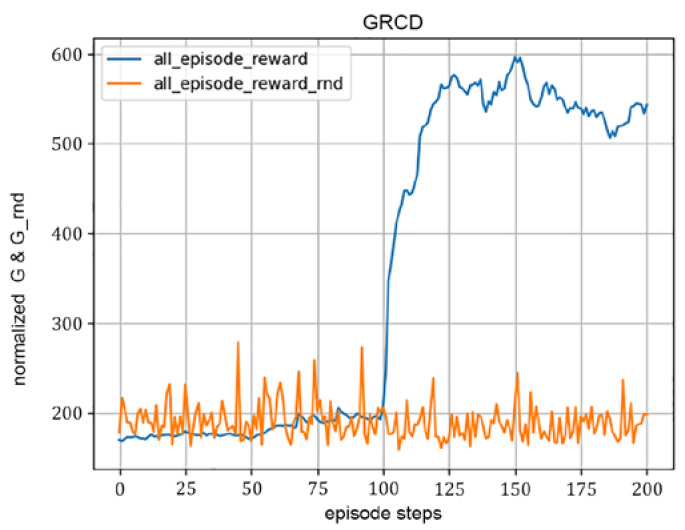
Convergence performance of the proposed algorithms with *N* = 49, Hidden_dim = 256.

This subsection presents a simulation of the convergence performance of the RIS-TD3 algorithm. The simulation conditions are as follows: the number of RIS reflection units is set to 49, the hidden layer dimensions of the Actor and Critic networks are set to 256, the number of exploration episode is 50, the agent-begins-to-learn episode is 100, and the total training episode is set to 200. We observe the cumulative reward of the episode obtained by the algorithm in each training episode, denoted as all_episode_reward, and apply a sliding average to it. Moreover, it is compared with the random RIS phase shift method, denoted as all_episode_reward_rnd. The simulation results are shown in Figure 4.

From Figure 4, it can be seen that the algorithm is in the random exploration and data accumulation phase before it starts learning (the first 100 rounds), and the accumulated episode reward obtained at this time is small and comparable to the average of the accumulated episode reward of the random RIS phase shift, which is within 200; when the algorithm starts to learn, the episode gain is able to increase rapidly, rising to a higher level of about 560 within about 25 episodes, and after that until the end of learning phase, the accumulated episode reward fluctuates in a smaller range, with the total gain above 500, which is much higher than that of the random RIS phase shifts. This demonstrates that the algorithm is able to be converged quickly and the rewards after convergence are greater, resulting in better convergence performance.

### 5.4. Computation Complexity Analysis

This subsection provides a time-consuming simulation for the complexity of the proposed algorithm. The benchmark algorithm used the RIS-DDPG algorithm, the SCA algorithm, and the SDR algorithm in the same way as in Section 5.2. The simulation condition is *N* = 36, the number of iterations for the SCA algorithm is 100, the iteration termination condition for the SDR algorithm is an error of less than 0.1, and the other simulation conditions are the same as in Section 5.2. Since both RIS-TD3 and RIS-DDPG can be learned offline and run online, we take the simulation time spent per episode in the testing phase after training as the algorithm consumption time. We recorded the time consumed by each algorithm for 10 simulations and averaged them to obtain the algorithm time-consuming simulation results shown in Table 2.

As can be seen from Table 2, the simulation time consumed by the RIS-TD3 algorithm proposed in the paper for one episode (200 steps) is comparable to that of the RIS-DDPG algorithm, with an increase of only 0.03 s. This is due to the fact that the proposed algorithm uses six DNNs while the RIS-DDPG algorithm only uses four DNNs. However, combining the results of 5.2, the RIS-TD3 algorithm obtains a median GRCD gain of 50 at *N* = 36 compared with that of RIS-DDPG, so the algorithm achieves a significant performance gain with a small time cost. Furthermore, the time consummation of the traditional SCA algorithm is 1.3 times longer than that of the proposed algorithm for only 100 iterations, but the average GRCD obtained is lower than that of the proposed RIS-TD3 algorithm. Finally, the traditional SDR algorithm needs the longest simulation time and has the lowest median GRCD performance. This indicates that the complexity of the proposed algorithm is significantly lower than that of the conventional algorithm.

### 5.5. Convergence Analysis

In this section, the hyperparameters’ effect on the RIS-TD3 intelligent signal detection algorithm’s performance is investigated. In the RIS-TD3, the main hyperparameters are as follows: the hidden layer size, exploration step size, discount rate, and learning rate. The effect of these hyperparameters on the RIS-TD3 algorithm’s performance is simulated. The other parameters and their corresponding values are as follows: the number of reflection units at RIS is 36, the length of batch learning data is 512, the discount rate is 0.99, the learning rate is 15 × 10^−5^, the hidden layer is 128, and the exploration step is 5000. The exploration noise gradually decreases when the empirical data are greater than two times the exploration step, with a decrease factor of 0.9999 until it decreases to 0.1.

**Figure 5:**
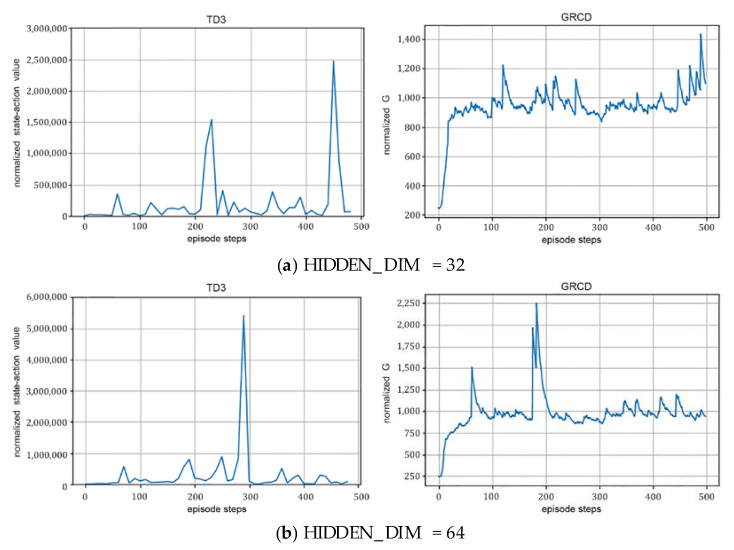

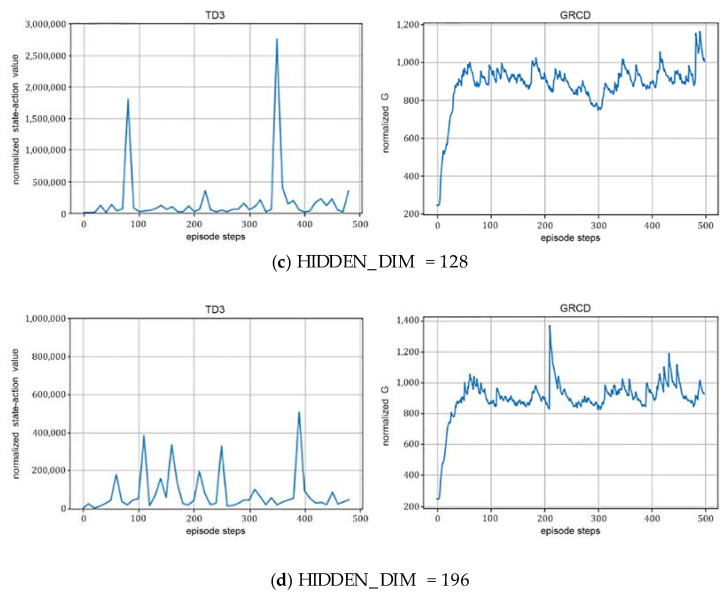
Effect of different hidden layer sizes on the algorithm performance with *N* = 36, γ=0.99.

(1) The hidden layer size:

As shown in Figure 5, as the hidden layer gradually increases from 32 to 64, the maximum cumulative reward value of rounds of the RIS-TD3 algorithm keeps increasing, and the average energy ratio of normalized rounds increases to over 1000. However, when the hidden layer is further increased to 128 or 196, RIS-TD3′s performance gradually decreases. This is because as the hidden layer increases, the DNN network is larger, and its learning and training complexity increase significantly, making it difficult to perform the training in the limited number of rounds. This suggests that the choice of hidden layers is a compromise between the system performance and algorithm complexity.

**Figure 6:**
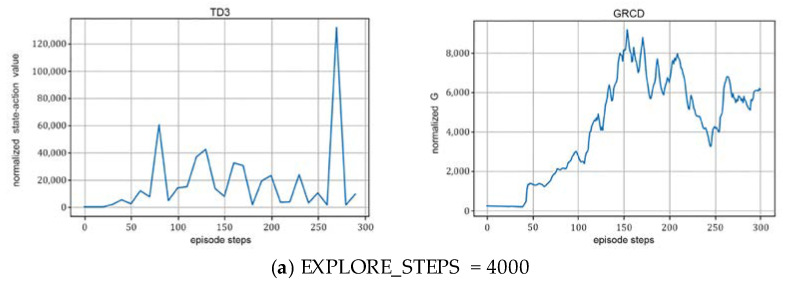

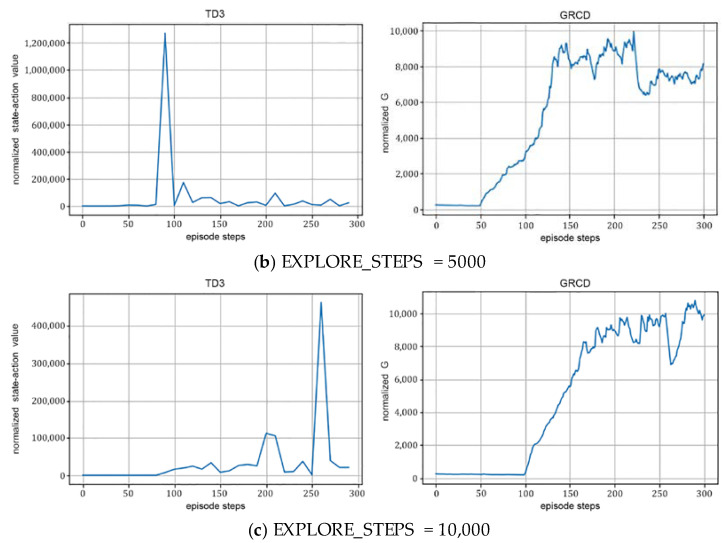
Effect of different exploration step lengths on the performance of the algorithm with *N* = 36, γ=0.99 and Hidden_dim = 128.

(2) The exploration step:

As illustrated in Figure 6, when the exploration step length is 4000, RIS-TD3 owes its first peak at around 80 rounds. As the iterations increase, RIS-TD3 owes its maximum peak at around 270 rounds, with a maximum peak of 131,703.35; when the exploration step length is 5000, RIS-TD3 has its maximum peak at around 90 rounds, with a maximum peak of 1,270,947.24. The optimal value can be achieved by RIS-TD3 quickly; when the exploration step length increases to 10,000, RIS-TD3 shows the first peak at 200 rounds and the maximum peak at 260 rounds, with a maximum peak of 463,016.65. According to the simulation results, it shows that as the exploration step length increases and more rounds are used, the later the DNN network starts learning, and the later the peak appears; however, with more exploration data as the initial sample for network learning, the faster the learning, and the higher the maximum peak can be achieved. When the exploration step is too large, the proposed RIS-TD3 algorithm cannot find the maximum value in the limited number of the iteration rounds, and the efficiency of the algorithm decreases.

**Figure 7:**
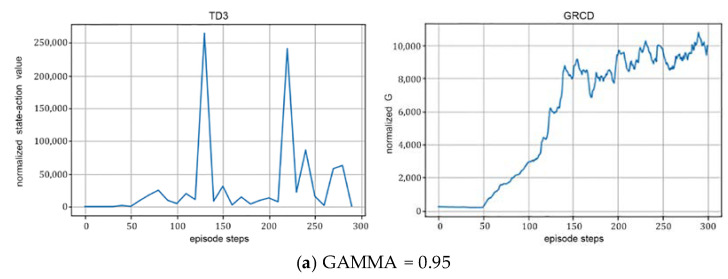

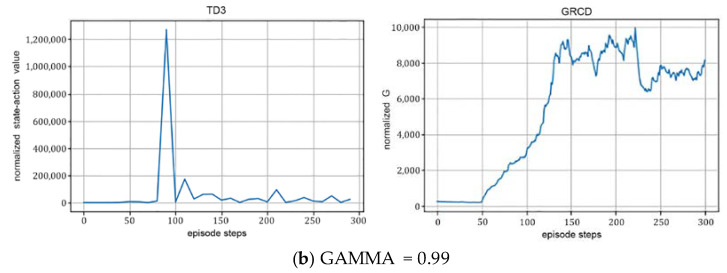
Effect of different discount rates on algorithm performance with *N* = 36, Explore_steps = 5000 and Hidden_dim = 128.

(3) The discount rate:

Figure 7 illustrates that when the discount rate is 0.95, RIS-TD3 owes the maximum value of 265,010.03 at round 140; when the discount rate increases to 0.99, the algorithm has the maximum value at round 90, and the maximum value reaches 1,270,947.24. This shows that the acquisition of the optimal value can be influenced significantly by the discount rate. The larger the discount rate is, the larger the reward of the algorithm can be achieved.

**Figure 8:**
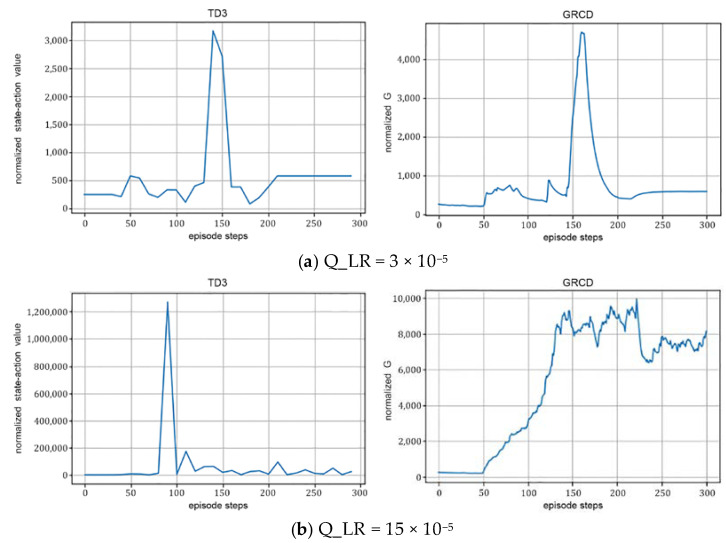
Effect of different learning rates on algorithm performance; *N* = 36, Explore_steps = 5000, γ=0.99, and Hidden_dim = 128.

(4) The learning rate:

As shown in Figure 8, when the learning rate is 3 × 10^−5^, RIS-TD3 peaks at round 140 with a peak value of 3174.04; when the learning rate is 15 × 10^−5^, the algorithm peaks at round 90 with a peak pick higher than that of the former. This indicates that the learning rate has significant influence on the optimal value, and when the learning rate is small, the proposed algorithm obtains a smaller optimal value.

## 6. Conclusions

In this paper, an RIS-assisted multi-antenna AmBC system model is investigated, and an efficient RIS-assisted AmBC signal detection method based on deep reinforcement learning, named RIS-TD3, is developed. Particularly, an RIS-assisted multi-antenna AmBC signal model is presented, which enables the information transmission and energy collection simultaneously. Furthermore, a Twin Delayed Deep Deterministic (TD3) signal detection method is built for the AmBC system. Compared with several state-of-the-art comparison methods, the presented RIS-TD3 method achieves better performance. Because of the powerful ability of deep reinforcement learning, more essential recessive features are well explored.

There are some important advantages about RIS-TD3: (1) RIS-TD3 has good stability by delay updating and smooth regularization of the target policy network, so as to obtain the optimal solution of the nonconvex optimization problem, lower bit error rate is obtained. (2) The effect of a hyperparameter on the performance of the algorithm is explored, and the hyperparameter is chosen appropriately to further improve detection performance. (3) RIS-TD3 does not need to estimate channel information in advance. (4) RIS-TD3 has lower bit error rate and more stable convergence performance than the reference method under the condition of lack of channel information. (5) The problems of incomplete parameter acquisition and serious direct link interference in traditional AmBC signal detection methods are avoided.

To further improve the signal detection performance, we will consider introducing hierarchical deep multi-task joint learning to learn more discriminative features efficiently. For model speed acceleration, a series of ingenious model compression technologies will be tried to combine into the deep reinforcement learning framework. Moreover, the effects of delicate adaptive self-training and practical unsupervised training schemes will be explored.

## Figures and Tables

**Table 1:** Simulation parameters.

Parameter	*Description*	*Value*
$\mu_{c}$	learing rate for q_net	0.00003
$\mu_{a}$	learing rate for policy_net	0.0003
$Int_{update}$	delayed steps for updating the policy network and target networks	3.0
$n_{explore}$	range of action noise for exploration	1.0
$n_{eval}$	range of action noise for evaluation of action value	0.5
D	buffer size for experience replay	500,000
I	the number of episodes	5000
T	the number of steps in each episode	20,000
W	the number of experiences in the mini-batch	64
γ	discounted rate for future reward	0.99
k	Rician factor	3
$d_{h}$	the distance from the BS to the IR	202 m
$d_{g}$	the distance from the RIS to the IR	30 m
$d_{H}$	the distance from the BS to the RIS	200 m

**Table 2:** Time consumed of various algorithms at N = 36 (unit: second).

Algorithms	RIS-TD3	RIS-DDPG	SCA	SDR
1	0.4188	0.3231	0.6420	10.4801
2	0.3837	0.3349	0.5787	7.0571
3	0.3684	0.3371	0.4785	7.5617
4	0.3647	0.3241	0.5332	9.7516
5	0.3868	0.4239	0.4490	5.9844
6	0.382	0.3531	0.4675	7.6004
7	0.369	0.3291	0.4493	6.6297
8	0.3786	0.3142	0.4527	6.0317
9	0.3526	0.3331	0.4411	7.2425
10	0.361	0.3577	0.5159	7.2832
Average	0.3766	0.3430	0.5008	7.5622
